# The *Sirt1* Gene Promotes Insulin Secretion in Accord with Diet

**DOI:** 10.1371/journal.pbio.0040044

**Published:** 2005-12-27

**Authors:** Françoise Chanut

Those seeking the fountain of youth would do well to watch their calories. Indeed, caloric restriction has been known for many years to increase longevity in laboratory animals. A simple explanation for this effect is that reducing food intake slows down the body's metabolism, which reduces the formation of toxic byproducts that cause tissue damage and aging.

Whatever the mechanisms, the benefits of caloric restriction hold from furry mammals to single-celled fungi. In yeast, nematodes, and fruit flies, caloric restriction increases the activity of the *Sir2* gene, which in turn changes the expression of genes related to metabolism. One of the key regulators of mammalian metabolism is the pancreatic hormone insulin. In a new study, Laura Bordone, Leonard Guarente, and their colleagues show that the mammalian homolog of *Sir2*, called *Sirt1*, modulates insulin production in response to diet.

In times of famine, the body taps into its own resources to provide energy for its working tissues. For instance, it mobilizes the lipid molecules stored in fat, and coaxes the liver into producing the simple sugar glucose. Cells take up glucose and lipids from the blood, and extract their chemical energy. In times of plenty, glucose and lipids come from food. As their levels rise in the blood, the pancreas secretes insulin, which stimulates the uptake of glucose by muscles and lipids by fat. An important function of insulin is to regulate glucose levels in the blood; its secretion is therefore tightly controlled by glucose concentration. But during fasting—and starvation—insulin secretion dips to very low levels, an adaptation that increases glucose availability for the brain.

Bordone et al. asked whether Sirt1 influenced insulin production. They disrupted the *Sirt1* gene of mice, and found that these mice produced very little insulin, regardless of whether they were well fed or starved. These results suggested that Sirt1 is necessary for glucose to induce insulin production.

The authors next asked at what step of insulin production Sirt1 acts. Insulin is made by specialized cells of the pancreas, called ß cells. ß cells can only secrete insulin when they accumulate enough ATP. This happens when glucose levels rise in the blood, after a meal for instance, because ß cells metabolize glucose into ATP. Bordone and her colleagues found that ß cells with an inactive *Sirt1* gene did not secrete as much insulin in response to glucose as normal ß cells. Nor did they convert glucose into ATP as efficiently as normal ß cells. This last observation led the authors to examine the activity of a type of protein known as uncoupling protein (UCP), which diverts glucose breakdown from ATP synthesis. In ß cells, the UCP2 protein is known to inhibit insulin secretion by routing glucose metabolism toward a molecule called NADH, rather than toward ATP.

The authors demonstrate that Sirt1 inhibits the production of UCP2 by directly preventing the expression of the *UCP2* gene. How does the interaction between Sirt1 and UCP2 relate to caloric restriction? The authors find that in starved mice, UCP2 levels increase in ß cells. This suggests that caloric restriction induces a decrease in Sirt1 activity in mice. This result is somewhat surprising since in yeast and other organisms, caloric restriction increases Sir2 expression.

Because Sirt1 and insulin have many roles in mammals, it is at present unclear how they mediate the effect of diet on lifespan. An intriguing hypothesis stems from the fact that UCP2 dampens the formation of toxic metabolic byproducts that precipitate aging. If the relationship between Sirt1 and UCP2 holds in more tissues than just ß cells, Sirt1 may open a simple path to a longer life.

